# Endophytic Bacterial Community Structure and Function of Herbaceous Plants From Petroleum Hydrocarbon Contaminated and Non-contaminated Sites

**DOI:** 10.3389/fmicb.2018.01926

**Published:** 2018-08-23

**Authors:** Rhea Lumactud, Roberta R. Fulthorpe

**Affiliations:** Department of Physical and Environmental Sciences, University of Toronto Scarborough, Toronto, ON, Canada

**Keywords:** bacterial endophytes, hydrocarbon degradation, plant growth promoting bacteria, oil field, stem endosphere, plant microbiome

## Abstract

Bacterial endophytes (BEs) are non-pathogenic residents of healthy plant tissues that can confer benefits to plants. Many Bacterial endophytes have been shown to contribute to plant growth and health, alleviation of plant stress and to in-planta contaminant-degradation. This study examined the endophytic bacterial communities of plants growing abundantly in a heavily hydrocarbon contaminated site, and compared them to those found in the same species at a non-contaminated. We used culture- dependent and independent methods to characterize the community structure, hydrocarbon degrading capabilities, and plant growth promoting traits of cultivable endophytes isolated from *Achillea millefolium, Solidago Canadensis*, and *Daucus carota* plants from these two sites. Culture- dependent and independent analyses revealed class Gammaproteobacteria predominated in all the plants regardless of the presence of petroleum hydrocarbon, with *Pantoea* spp. as largely dominant. It was interesting to note a >50% taxonomic overlap (genus level) of 16s rRNA high throughput amplicon sequences with cultivable endophytes. PERMANOVA analysis of TRFLP fragments revealed significant structural differences between endophytic bacterial communities from hydrocarbon-contaminated and non-contaminated soils—however, there was no marked difference in their functional capabilities. *Pantoea* spp. demonstrated plant beneficial characteristics, such as P solubilization, indole-3-acetic acid production and presence of 1-aminocyclopropane-1-carboxylate deaminase. Our findings reveal that functional capabilities of bacterial isolates being examined were not influenced by the presence of contamination; and that the stem endosphere supports ubiquitous BEs that were consistent throughout plant hosts and sites.

## Introduction

Plants form associations with a multitude of structurally and functionally diverse beneficial microorganisms that can provide them with selective advantages. Among these beneficial associates are bacterial endophytes (BEs) - non-pathogenic bacteria that reside within the living tissues of plants without conferring them harm. Many BEs have been reported to support growth, improve plant health and alleviate stress (Azevedo et al., 2000; Hardoim et al., 2008; Glick and Stearns, 2011; Mitter et al., 2013).

A growing body of literature demonstrates evidence of some BEs' ability to mineralize petroleum hydrocarbon components (Phillips et al., 2008, 2009; Andria et al., 2009; Yousaf et al., 2010, 2011; Afzal et al., 2012; Kukla et al., 2014). Notably, Yousaf et al. (2010, 2011) found endophytic strains of *Enterobacter ludwigii* and *Pantoea* could successfully colonize plants Italian ryegrass and birdsfoot trefoil, and therein mineralize hydrocarbon, and express genes for hydrocarbon degradation. Improved contaminant degradation was correlated with increased numbers of pollutant-degrading bacteria (Yousaf et al., 2011).

A study on the bacterial epiphytes and their potential to bioremediate hydrocarbon pollutants in the atmosphere revealed significantly higher hydrocarbon degrading epiphytes isolated from a polluted environment compared to those isolated from a pristine environment (Ali et al., 2012). Oliveira et al. (2014) reported that the level of hydrocarbon pollution in salt marsh sediments was the determining factor in endophytic community composition, however, it is still unclear if this holds true for stem endophytic bacteria from plants in grassland or terrestrial systems.

In contaminated environments, BEs can also help plants tolerate contaminant-induced stress by releasing 1-aminocyclopropane-1-carboxylate (ACC) deaminase that decreases ethylene production (Glick, 2004). Some BE strains produce indole-3- acetic acid and solubilize inorganic phosphates, thereby promoting plant growth (Sheng et al., 2008; Dashti et al., 2009; Becerra-Castro et al., 2011). Recently, the proposal that BEs possessing both contaminant-degrading and plant growth promoting capabilities would be more likely to succeed in cleaning up organic contaminants recognizes increasing need for this research (Khan et al., 2013; Afzal et al., 2014; Kukla et al., 2014).

Oil Springs, Ontario, Canada (N42°46. 267, W82°05.539), sits above a naturally occurring near surface oil deposit. Oil has been seeping to the surface and forming gum patches since human recorded history in the area. Hand-dug wells and pumps were established as the 1850's and the pumps still produce oil to this day. During pump services, oil frequently spills onto the nearby soils leading to total petroleum hydrocarbon concentrations in the spill areas from 45,000–300,000 ppm. Despite the known toxicity of petroleum hydrocarbon, several common plant species grow abundantly in these soils.

Plants naturally produce alkanes, aromatic hydrocarbons and other compounds that share structural similarities with many organic pollutants; and some of these compounds are intermediates of degradation pathways that are produced during catabolism of organic contaminants. Bacteria that are in intimate association with plants are known to consume plant exudates, and are deemed capable in degrading organic contaminants. Hence, the role of plant bacterial partners in remediating soils contaminated by organic compounds has been well documented (Weyens et al., 2009; Vangronsveld et al., 2011; Khan et al., 2013; Afzal et al., 2014; Gkorezis et al., 2016; Ijaz et al., 2016).

For all these reasons, we hypothesized that endophytic bacterial communities of these Oil Springs plants might be contributing significantly to their adaptation to petroleum hydrocarbons (PHCs) toxicity. We predicted that (1) these plants harbor high numbers of endophytic bacteria that are able to mineralize PHC and (2) these plants harbor high numbers of endophytic bacteria with plant growth promoting capabilities. To this end we compared the endophytic bacterial populations found in herbaceous species thriving at Oil Springs to those found in the same species growing at a control, uncontaminated site.

## Materials and methods

### Site description and sampling

The hydrocarbon contaminated site is a natural oil seep field located at Oilsprings, Ontario (N42°46. 267, W82°05.539). Soils have silty clay loam soil texture, with pH of 6.8–7.3, and extractable total P and total N were 21 mg/L and 0.44% dry soil, respectively. The non-hydrocarbon contaminated site is located around 80 km away at Komoka, Ontario (N42°56.850, W81°23.697), which is an uncontaminated meadow with undetectable levels of PHCs. The soil was silty loam soil, a pH of 7.7 and extractable total P and total N of 5.3 mg/L and 0.20% dry soil, respectively.

Three pumping wells with recurring spillage were randomly chosen as sampling locations across a 650-acre oil field; whereas in non-contaminated site, three random sampling locations were chosen across a 500 m transect line. Three plant species belonging to—*Achillea millefolium, Solidago canadensis*, and *Daucus carota* (at least five individual plants of each plant species to minimize spatial individual plant variation), of the same size and maturity, were sampled. These plants were chosen as these were seen growing at both the hydrocarbon and non-hydrocarbon contaminated sites. All plants were immediately placed in Ziploc bags and into a 4°C cooler box for transport to the lab.

### Culture-based analysis: endophyte isolation and identification

In each replicate, 15–20 g of stem tissues were surface- sterilized using a series of washes (70%ethanol; 1.2%bleach with 0.1% Tween 20; followed by six washes of sterile distilled water). To test the efficacy of sterilization, an aliquot of the last wash was plated onto agar plates and sterilized stem samples were imprinted onto both Reasoner's 2A (R2A) and Tryptic Soy Agar (TSA) media. The sterilized stems were then macerated in a sterilized Waring blender vessel at 2,0000 rpm using sterile 60 ml 50 mM Tris-HCl and heterotrophic bacteria were isolated by plating 100 μL on R2A and TSA plates. The media plates were incubated at 28°C for a period of 1–4 weeks. Individual bacterial colonies were isolated and grown into pure culture. Lysates were made from pure colonies by boiling 2 loopfuls of 1 μL sterile disposable loops in 100 μL sterile distilled water for 7 min. One microliter of lysate was used as template in a PCR reaction using 16SrRNA primers 27F (5′-AGAGTTTGATYMTGGCTCAG-3′) and 1492R (5′-TACCTTGTTACGACTT-3′; Frank et al., 2008). The PCR reaction was as follows: 20μL reactions with a final concentration of 0.5 mM of the forward primer and reverse primer, 1.5 mM MgCl2, 200 mM of each dNTP, 2.5 units of HotStarTaq Plus DNA polymerase (Qiagen, Canada). The PCR amplifications were carried out in a PTC-200 thermal cycler (MJ Research Inc.) with the following conditions: initial denaturing at 95°C for 5 min followed by 35 cycles of: denaturing at 95°C for 1 min, annealing at 56°Cfor 1 min and extension at 72°C for 1 min; final extension at 72°C for 10 min. The resultant amplicons were purified and subsequently submitted for Sanger sequencing at The Centre for Applied Genomics (TCAG) sequencing facility (Toronto, Canada) using 27F (5′-AGAGTTTGATYMTGGCTCAG-3′) primer. The identity of the isolates was determined using the most similar 16S rDNA sequences with the Ribosomal Database Project; sequences were deposited in GenBank with accession numbers: MH470404 - MH470471.

### Screening for hydrocarbon degrading potential of bacterial endophytes

Mineralization of hydrocarbon by individual endophytes was quantified using rapid growth based colorimetric assays and also via PHC loss measurements in liquid cultures via Gas Chromatography-Flame Ionization Detector (GC-FID). These methods are detailed elsewhere (Lumactud et al., 2016).

Isolates were screened for the presence of known catabolic genes for enzymes: alkane hydroxylase (AlkB), using primers alkBwf 5′-AAYAC NGCNCAYGARCTNGGVCAYAA-3′ and alkBwr 5′-GCRTGRT GRTCHGARTGNCGYTG-3′ that targets groups belonging to *Acinetobacter, Pseudomonas* and *Rhodococcus* (Wang et al., 2010). PCR conditions were initial denaturing at 94°C for 4 min followed by 32 cycles of: denaturing at 94°C for 30 s, annealing at 55°C for 30 s and extension at 72°C for 1 min; final extension at 72°C for 10 min. Catechol 2,3-dioxygenase (C23O) genes were assayed using primers C23O-F-AGGTGCTCGGTTTCTACCTGGCCGA and C23O-R-ACGGTCATGAATCGTTCGTTGA G (Luz et al., 2004) using PCR conditions- initial denaturing at 94°C for 4 min followed by 30 cycles of: denaturing at 94°C for 1 min, annealing at 60°C for 1 min and extension at 7°C for 1 min; final extension at 72°C for 3 min. Primers that were used for cytochrome P450-type alkane hydroxylase gene assay were F- GTSGGCGGCAACGACACSAC and R- GCASCGGTGGATGCCGAAGCCRAA, following the conditions described in (Arslan et al., 2014). Catechol 1, 2 dioxygenase genes assays were done using the primers—cat1,2-F-ACVCCVCGHACCATYGAAGG and cat1,2-R- CGSGTNGCAWANGCAAAGT following the PCR conditions as described elsewhere (El Azhari et al., 2010).

### Evaluation of plant growth promoting abilities and 1-aminocyclopropane-1-carboxylate deaminase gene (ACCD)

All isolates were assessed for production of indole-3-acetic acid (IAA), solubilization of inorganic phosphate and the presence of the 1-aminocyclopropane-1-carboxylate deaminase gene. IAA production by bacterial isolates both in the presence and absence of L-tryptophan (L-TRP) was measured following the method described by Gordon and Weber (1951). The phosphate solubilization ability of the isolates was determined on a Pikovskaya agar medium. The presence of a clear zone around the bacterial colonies indicates the solubilization of phosphate. The halo size was calculated by subtracting the colony diameter from the total diameter. This assay was done in duplicate. 1-aminocyclopropane-1-carboxylate deaminase gene (*acdS*) was assayed following Blaha et al. (2006).

### Culture-independent community analysis

Total community DNA was extracted from plant macerates using FastDNA SPIN Kits (MP Biomedicals) following manufacturer's instructions with modifications (addition of 100 μL of protein precipitation solution solution to the lysing solution and two additional SEW-S washes of the bound DNA). Community structure and taxonomic diversity were examined using Terminal Restriction Fragment Length Polymorphism (TRFLP) of PCR-amplified 16S rRNA gene fragments. The genomic DNA was initially amplified with universal bacterial primers 27F (5′-AGAGTTTGATYMTGGCTCAG-3′) and 1492R (5′-TACCTTGTTACGACTT-3′) using conditions as above. The resultant amplicons were digested with restriction enzymes PvuII and MscI (NEB Canada) to minimize amplification of chloroplasts and mitochondria (Shen and Fulthorpe, 2015). One microliter of the resultant digested product was used as template in PCR reaction (same conditions as above) using 16S rRNA fluorescein labeled primers, 27F-FAMand 1492R-HEX (LifeTechnologies, Canada). The generated amplicons were digested with restriction enzyme MspI and sent to the Agriculture and Food laboratory at the University of Guelph for fragment analysis.

FAM labeled terminal fragments were used to determine phylotype densities and richness after fragments <60 bp in size were omitted in the analysis. The Microsoft Excel macro Treeflap (Rees et al., 2005), obtained from http://urbanstreams.net/index.php/the-treeflap-macro/, was used to round the fragment sizes to the nearest one base pair and to align the fragments of the same size from different samples. The height data was converted to % abundance based on total fluoresence and any fragments that represented <1% abundance were omitted.

### 16s rRNA gene sequencing analysis

DNA amplification of the16s rRNA gene sequencing of triplicate pools of samples (6 samples) was performed at Molecular Research LP (Shallowwater, Texas, USA) on an illumina MiSeq following the manufacturer's guidelines. The 16s rRNA gene V4 hypervariable region was amplified using the PCR primers 515/806 with barcode on the forward primer. PCR reactions were prepared using the HotStarTaq Plus Master Mix Kit (Qiagen, USA) under the following conditions: 94°C for 3 min, followed by 28 cycles of 94°C for 30 s, 53°C for 40 s and 72°C for 1 min, after which a final elongation step at 72°C for 5 min was performed. 16s sequence data were trimmed, denoised, and chimera depleted with default parameters using Qiime pipeline v.1.8 (Caporaso et al., 2010). 16S rRNA taxonomy was assigned using RDP classifier trained using the greengenes input files provided by Qiime (DeSantis et al., 2006). Raw sequencing data can be retrieved from the Short Read Archive under the study accession- PRJNA475746.

### Data analyses

Analysis of Variance tests (ANOVAs) were used to compare total culturable heterotrophic bacteria and phylotype richness of each plant species, thereafter, a *post-hoc* test for was done using bonferroni correction. All the above-mentioned data analyses and graphs were made using Microsoft excel. A non-metric multidimensional scaling (NMDS) based on Bray Curtis dissimilarities was used for the ordination of TRFLP dataset. Permutational ANOVA was then carried out using the function Adonis in the vegan package (Oksanen et al., 2012) for R studio (RStudio Team, 2015).

## Results

Endophytic bacterial communities were isolated from the stem endosphere of *Daucus carota* (wild carrot), *Achillea millefolium* (yarrow) and *Solidago canadensis* (goldenrod). Community characterizations were done using culture- dependent and independent means. Phenotypic characterization assays were done on the culturable fraction of endophytic bacterial strains.

### Community characterizations

#### Culturable communities

**Figure 1:**
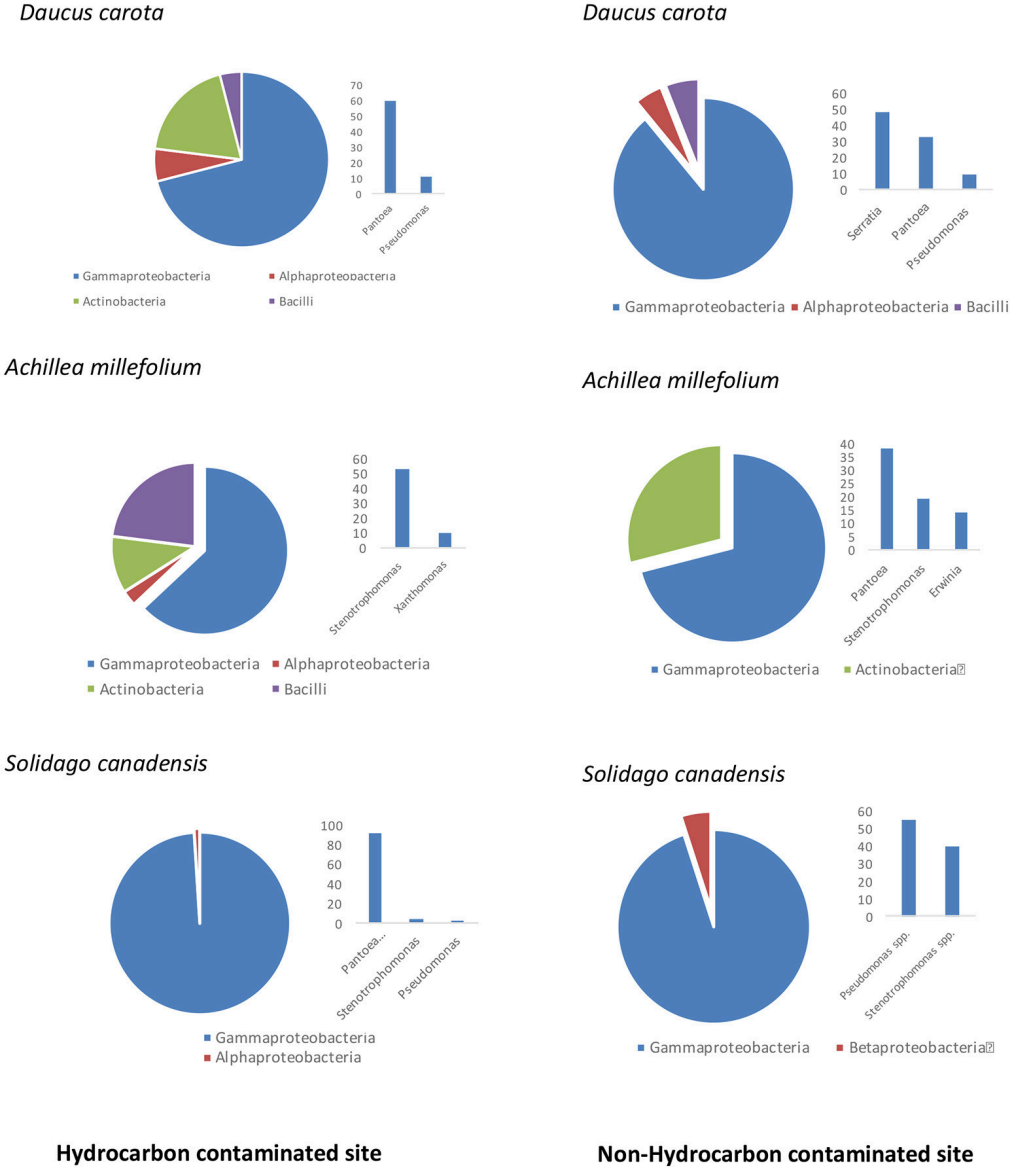
Comparison of culturable endophytic bacterial communities isolated from three plant species growing in hydrocarbon contaminated and non-hydrocarbon contaminated sites.

**Figure 2:**
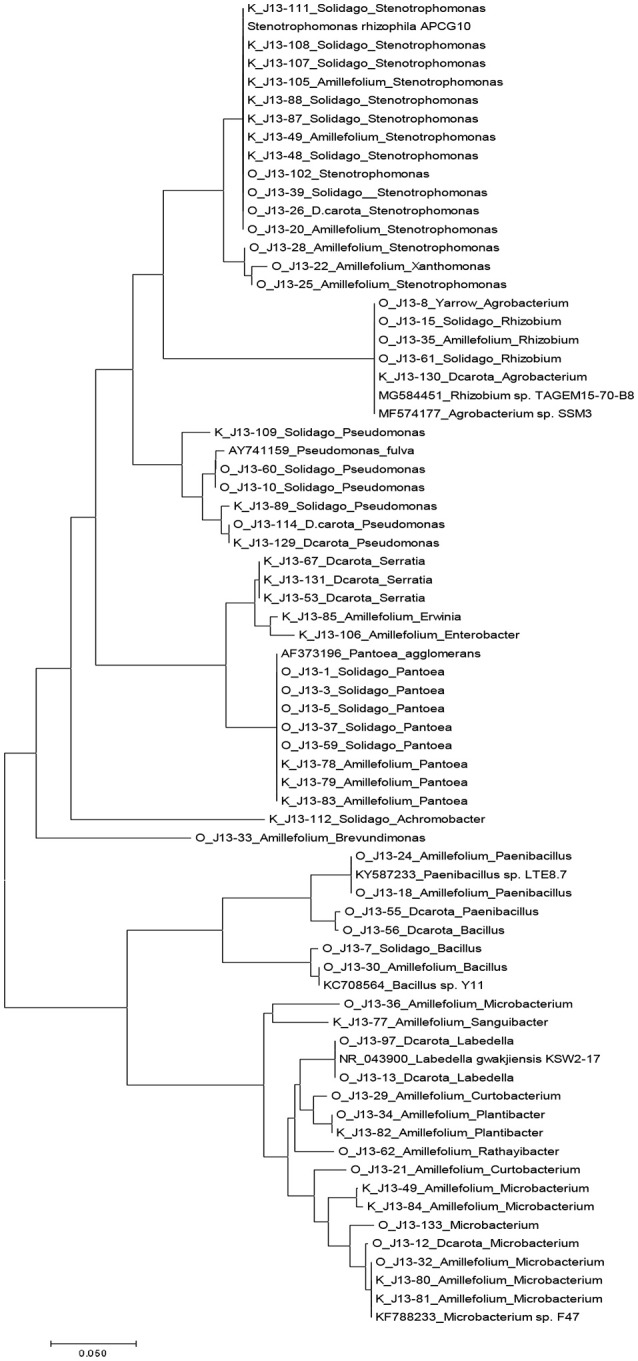
Phylogenetic tree based on partial 16s sequences inferred using the Neighbor-Joining method. The evolutionary distances were computed using the maximum composite likelihood method in MEGA7 (Kumar et al., 2016). Strains that do not have J13 labels are sequences from Genbank database.

An average of ~2000 CFU/g of fresh stem tissue were recovered from the plants. There were no significant differences of culturable endophytes abundance and TRFLP phylotype richness (Table 1). Figure 1 illustrates the culturable endophytic bacterial communities isolated from three plant species in hydrocarbon (HC) and non-HC contaminated sites. Gammaproteobacteria were predominant in all the plants and at both sites. Additional phyla were recovered from the plants growing in HC contaminated site. An additional phylum (Actinobacteria) was recovered from wild carrot at the contaminated site. The phyla Alphaproteobacteria and Firmicutes, absent in yarrow from non-HC contaminated site, were retrieved from yarrow in HC contaminated site. Gammaproteobacteria and Alphaproteobacteria were recovered from goldenrods in HC contaminated site while Gammaproteobacteria and Betaproteobacteria were recovered in non-hydrocarbon contaminated site. Similar predominant taxa were recovered in each plant species regardless of contamination. *Pantoea* and *Pseudomonas* spp. dominated the culturable flora of the wild carrot plants from both sites. *Stenotrophomonas* spp. were recovered in yarrow plants from both sites, whereas, both *Pseudomonas* and *Stenotrophomonas* were recovered from goldenrods at both sites. *Pantoea* spp., having been retrieved from all the plants regardless of contamination seemed to be ubiquitous. Phylogenetic structure of the 16s rRNA revealed grouping of cultivable bacterial endophytes regardless of plants host species and contamination (Figure 2).

**Table 1:** Mean values (*N* = 3) of colony forming units and species richness using TRFLP fragments of endophytic bacterial communities per gram fresh weight of plant tissues recovered from *Daucus carota, Achillea millefolium*, and *Solidago canadensis* from petroleum hydrocarbon (HC) contaminated and non-contaminated sites, standard deviation in parenthesis.

	**Plant species**	**CFU/g**	**Phylotype richness (TRFLP fragments)**
HC contaminated site	*Daucus carota*	2620 (1015)^a^	10 (1.4)^a^
	*Achillea millefolium*	1667 (1193)^a^	12.7 (2.3)^a^
	*Solidago canadensis*	1960 (1061)^a^	12 (1)^a^
NON-HC contaminated site	*Daucus carota*	1233 (929)^a^	11.7 (2.9)^a^
	*Achillea millefolium*	2200 (1386)^a^	12 (2)^a^
	*Solidago canadensis*	2433 (404)^a^	12.7 (2.1)^a^

*Means with different letters are significantly different at P < 0.05*.

#### Culture-independent communities

**Figure 3:**
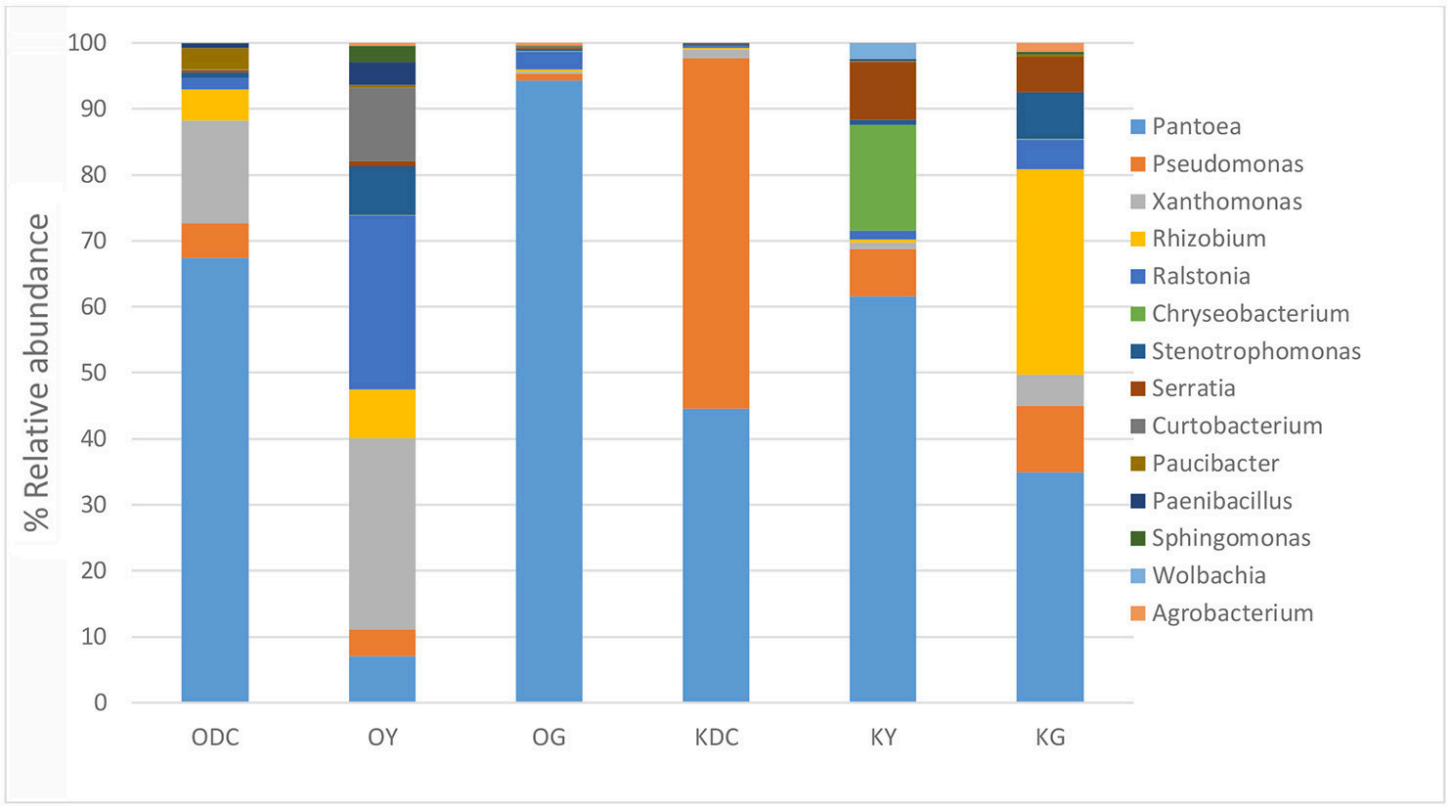
Analysis of culture-independent stem endophytic communities at genus level isolated from plants growing in hydrocarbon contaminated (sample name starts with O for Oil Spring) and non-hydrocarbon contaminated soils (sample name starts with K for Komoka)—ODC, Oil Spring Daucus carota; OY, Oil Spring yarrow (*A. millefolium*); OG, Oil Spring goldenrod (*S. canadensis*); KDC, Komoka Daucus carota; KY, Komoka yarrow; KG, Komoka goldenrod.

**Figure 4:**
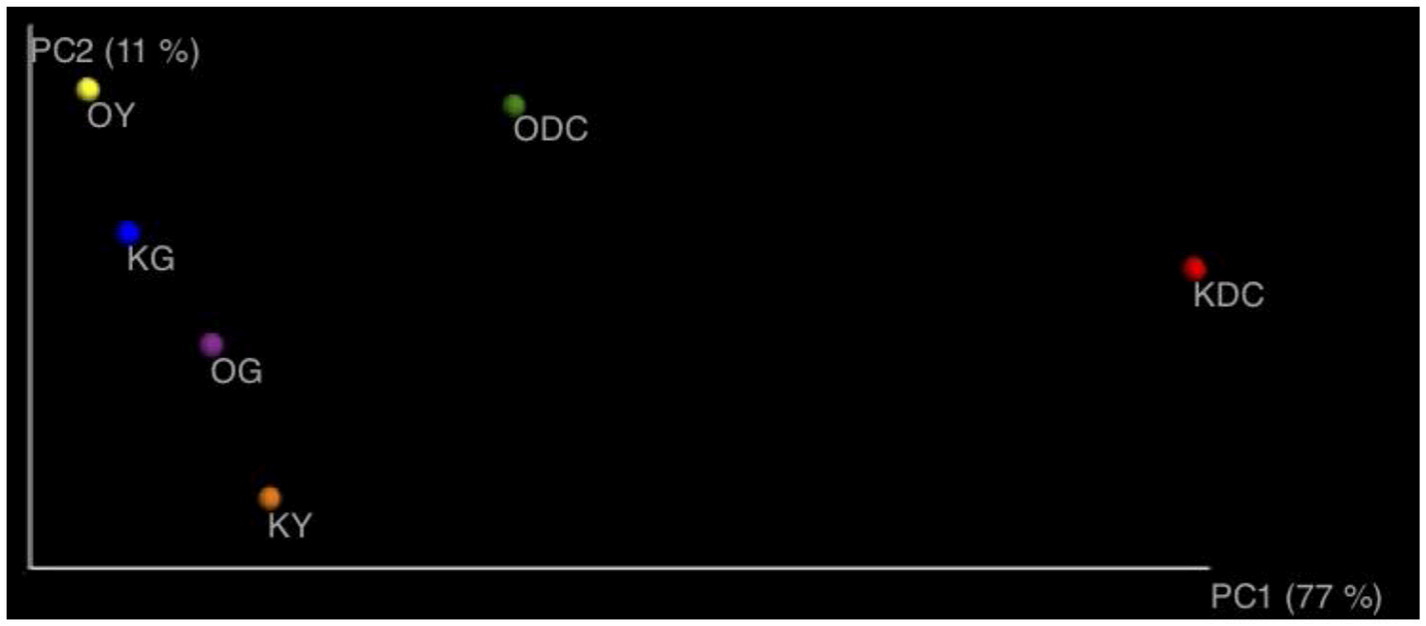
Principal coordinate analyses of axes 1 and 2 using weighted unifrac distances.

Figure 3 illustrates 16s rRNA amplicon sequencing showed Gammaproteobacteria to dominate all the samples in all the plant species and at both sites. *Pantoea* predominated all the plants at both sites except in yarrow plants at HC contaminated site where they were predominated by *Ralstonia* and *Xanthomonas*. At non-HC site, *Daucus carota* were also predominated by *Pseudomonas*, while *Rhizobium* predominated the goldenrods. Alpha diversity indices are presented on Table 2 with non-HC samples showing, on average, a generally lower indices. Principal coordinate analyses of weighted unifrac distances as an indication of beta diversity revealed that irrespective of petroleum hydrocarbon contamination of the site, the bacterial communities in the stem endosphere of *Daucus carota* plants were different from the rest of the plant samples (Figure 4).

**Table 2:** Alpha-diversity.

	**Sample**	**Observed**	**Shannon**	**Chao1**
Hydrocarbon-contaminated sites	OG	113	1.9	239
	OY	270	3.2	481
	ODC	237	3.2	442
Non-hydrocarbon-contaminated sites	KG	201	3.0	297
	KY	141	2.6	279
	KDC	123	2.7	172

**Figure 5:**
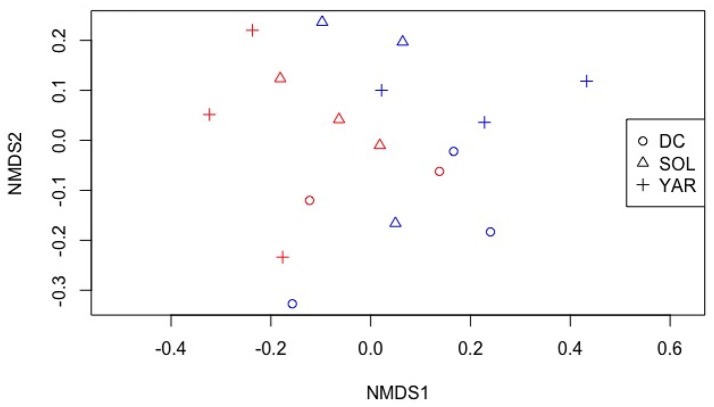
Non-metric multidimensional scaling of TRFLP profiles of bacterial endophytic communities from different plant species. Stress: 0.14. DC- Daucus carota (Wild carrot) SOL- Solidago canadensis (Goldenrod) YAR- Achillea millefolium (Yarrow). Red color- contaminated site Blue color- non-contaminated site.

We examined the endophytic community assemblages using nonmetric multidimensional scaling of TRFLP fragments as shown in Figure 5. Though there was no visually distinct grouping of plant species and location, permutational multivariate analysis of variance revealed significant differences of endophytic bacterial phylotypes of differing plant species (*P* < 0.05, F model = 1.78, *R*^2^ = 0.45). There was also a significant interaction effects of plant species and location (*P* < 0.05, F model = 2.21, *R*^2^ = 0.12).

### Functional characterizations

A total of 99 isolates, of which 54 isolates (*Solidago*- 16 *Daucus*- 14 *Achillea*- 24 and 45 (*Solidago*- 16 *Daucus*- 15 *Achillea*- 14) from HC contaminated and non-HC contaminated sites, respectively, were assayed for their hydrocarbon degradation capabilities, production of indole acetic acid, P solubilization, presence of hydrocarbon degrading and ACC deaminase genes.

**Figure 6:**
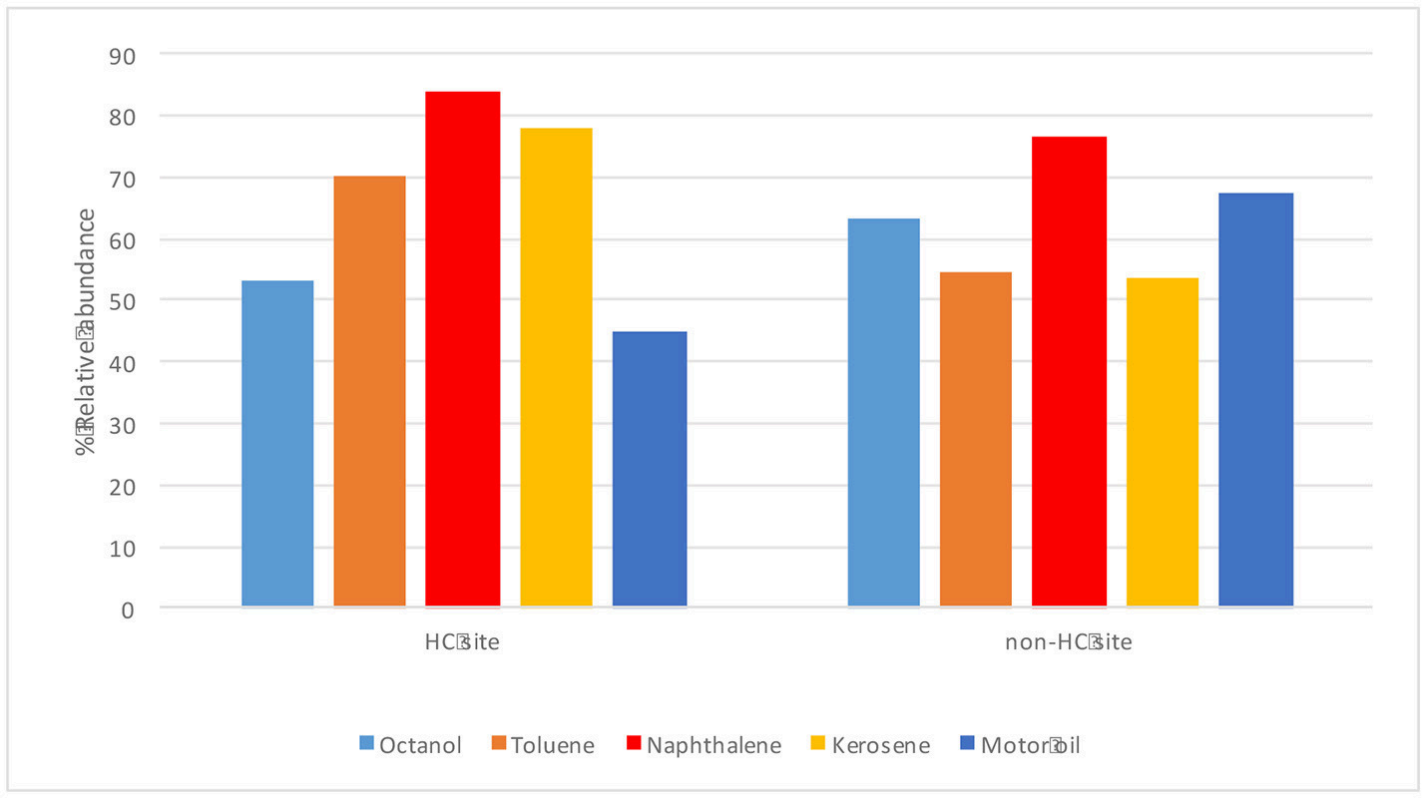
Hydrocarbon degrading potential (through colorimetric mineralization assay) of different petroleum hydrocarbon substrates by endophytic bacterial isolates from hydrocarbon contaminated and non- hydrocarbon contaminated sites.

**Figure 7:**
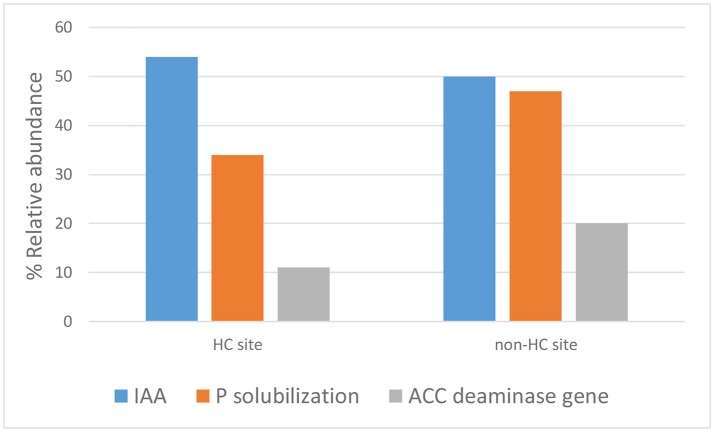
Plant growth promoting and stress resistance potential of bacterial endophytes isolated from hydrocarbon contaminated and non- hydrocarbon contaminated site.

The endophytes isolated from non-HC contaminated site did not show marked difference from those isolated from HC contaminated site in their hydrocarbon degradation ability on various petroleum hydrocarbon substrates (Figure 6). Catechol 2,3-dioxygenase and P450 genes were not detected in any of the isolates. Figure 7 shows plant growth promoting and stress resistance capabilities of the bacterial endophytes. Results show that 54% and 50% of isolates from HC and non-HC contaminated sites, respectively, were able to produce indole acetic acid in the presence of tryptophan. The endophytes were also able to solubilize inorganic phosphate into soluble form. Results revealed higher number of percent relative abundance of P solubilization ability in non-HC site at 47% compared to 34% from HC site. The ACC deaminase production potential of the isolates was evaluated through presence of *acdS* gene, 11% of the isolates tested from HC contaminated site possessed acc deaminase genes, while 20% from non-HC contaminated site. Table 3 shows the results of the functional characterization of bacterial endophytic strains grouped according to plant species and location. We also showed mineralization activity of bacterial endophytes on toluene and naphthalene using GC-FID method; it is interesting to note that there was no difference in mineralization pattern between HC contaminated and non-HC contaminated sites.

**Table 3:** Plant growth promoting and hydrocarbon degrading potentials of bacterial endophytes isolated from hydrocarbon and non-hydrocarbon contaminated sites.

**Site**	**Plant**	**Closest type strain**				**Hydrocarbon degrading genes**		**GC FID mineralization activity**	
			**P solubilization**	**IAA**	**ACC deaminase gene**	***alkB***	**Cat1,2**	**Toluene**	**Napthalene**
Hydrocarbon contaminated site	*Achillea millefolium*	*Agrobacterium tumefaciens*	–	–	–	–	–	–	4.6
		*Bacillus* spp.	±	±	–	–	–	±, 9.5	–
		*Curtobacterium* spp.	–	+	–	–	–	–	–
		*Microbacterium oxydans*	+	–	–	–	–	–	–
		*Paenibacillus illinoisensis*	–	+	–	–	–	na	na
		*Paenibacillus* sp	++	–	–	–	–	na	na
		*Plantibacter flavus*	++	+	–	–	–	–	–
		*Rhizobium* sp	–	–	+	–	–	–	–
		*Stenotrophomonas chelatiphaga*	–	–	–	+	–	–	–
		*Stenotrophomonas* sp.	–	–	–	+	–	12.7	–
		*Xanthomonas campestris*	–	–	+	–	–	–	–
		StrainJ13–19	–	–	–	–	–	86.2	–
		StrainJ13–31	–	+	–	–	–	22	–
	*Daucus carota*	*Microbacterium testaceum*	+	+	–	–	–	–	–
		*Paenibacillus amylolyticus*	–	±	–	–	–	±, 9.2	–
		*Pantoea agglomerans*	+	+	±	–	–	–	–
		*Labedella gwakjiensis*	na	±	–	–	–	–	–
									
	*Solidago canadensis*	*Pantoea agglomerans* spp.	+	±	–	–	–	±, 3.8	–
		*Pseudomonas fulva*	–	+	–	–	–	–	–
		*Pseudomonas* st J13–60	–	+	+	–	+	9.2	–
		Stenotrophomonas spp.	–	+	±	–	–	–	–
		StrainJ13–38	+++	+	+	–	–	–	–
Non–hydrocarbon contaminated site	*Achillea millefolium*	*Erwinia*	+++	+	+	–	–	–	–
		*Microbacterium hydrocarbonoxydans*	–	–	–	–	–	4.3	–
		*Microbacterium hydrocarbonoxydans* J13–84	–	+	–	–	–	–	–
		*Pantoea agglomerans* J13–78	+	–	+	–	–	–	–
		*Pantoea agglomerans* J13–79	+	+	+	–	–	69.6	48.2
		*Pantoea agglomerans* J13–83	++	+	–	–	–	–	–
		*Plantibacter cousiniae*	–	+	–	–	–	–	–
		*Sanguibacter inulinus*	–	–	–	–	–	24.4	20.4
		*Stenotrophomonas rhizophila*	–	–	–	–	–	19.0	16.7
	*Daucus carota*	*Pantoea agglomerans* J13–52	+	–	–	–	–	–	–
		*Pantoea agglomerans* J13–66	+	+	–	–	–	19.4	9.8
		*Pseudomonas putida* str 129	na	na	+	+	+	–	–
		*Pseudomonas* str J13–114	+	+++	–	–	+	–	–
		*Serratia ficaria*	+	–	–	–	–	–	–
		*Serratia plymuthica*	+	+	–	–	–	–	–
		Strain J13–74	+	+++	+	–	–	–	–
		Strain J13–76	+++	–	–	–	–	22.7	17.5
	*Solidago canadensis*	*Achromobacter* sp.	+	+					
		*Pseudomonas putida* J13–89	+	+++	+	–	+	–	–
		*Pseudomonas* str J13–109	+	–	+	–	–	–	–
		*Stenotrophomonas maltophilia*	–	±	–	–	–	–	–
		*Stenotrophomonas rhizophila*	–	±	–	–	–	–	–
		*Stenotrophomonas* str J13–108	–		+	–	–	–	–

*na, not assayed; -, not detected or no significant loss under GC-FID mineralization acitivity. ±, indicates that some isolates being tested were negative. +, means positive for the assay; ++, strongly positive; +++, very strongly positive*.

## Discussion

To the best of our knowledge, this is the first report that looked at community composition, structure and function of BEs in pioneer plants growing in both chronically contaminated with high levels of petroleum hydrocarbon and non-hydrocarbon contaminated sites. Moreover, this study looked at both the culture- dependent and independent endophytic bacteria in the stem endosphere, a largely understudied ecological niche in plant-bacterial system. Since root endophytes are highly derived from the rhizosphere, we focused on endophytes present in stems tissue as we are most interested in those that are selected by the plant and not the soil ecosystem. The plants are not only exposed to both soluble HC from the soil, but also to volatile HC from atmosphere at the site. The study of the stem microflora was assumed to not only be reflective on both these selection pressures from HC contamination but also of adaptation to the plant interior.

Our results show significant differences in community composition, through TRFLP fragments, across plant species and plant host-contamination interaction. This finding was in agreement with the previous studies where bacterial community structure differs with presence of contaminants and differing hydrocarbon levels at sites with simulated contamination (Phillips et al., 2009; Afzal et al., 2011; Kukla et al., 2014). PCoA of 16s amplicon sequencing showed separation of *D. carota* stem endophytes from the rest of the samples. It is interesting to note that *D. carota* plant belongs to family Apiaceae, while *A. millefolium*, and *S. canadensis* belong to family Asteraceae. Although inconclusive, it seems that plant host families influence bacterial diversity of the stem endosphere.

For both culture-dependent and independent community composition, we found that Gammaproteobacteria, particularly those genera from family Enterobacteriacea dominated the plant endospheric communities regardless of plant host species and contamination. Genera from family Enterobacteriaceae was also found to predominate the endosphere rather than the rhizosphere of plants in Athabasca oilsands reclamation sites (Mitter et al., 2017). Gammaproteobacteria was also reported to be predominant in the endosphere of ginseng plants (Khan Chowdhury et al., 2017). Except for *Achillea millefolium* in contaminated site, which was dominated by *Xanthomonas* and *Ralstonia, Pantoea* were abundant in all the plants. The *Pantoea* sp. in this study were closely related to *P. agglomerans* and *P. vagans*. *P. vagans*, which was formerly reported as *P. agglomerans* and *Erwinia herbicola*, is a common plant epiphyte and has been reported to control fire blight caused by the related enterobacterium *Erwinia amylovora* (Smits et al., 2010). *P. agglomerans* was reported as a potential plant growth promoting endophytic diazotroph for deep water rice (Verma et al., 2001) and many other plants. In this present study, *Pantoea* spp. demonstrated production of IAA and phosphorus solubilization.

It was interesting to discover a >50% overlap (genus level) of culture-independent and cultivable endophytic bacterial community structure. As shown in Figures 1, 3, predominating culture-dependent bacterial endophytes were also predominant using culture-independent techniques, revealing high cultivability of these BEs. Table 4 shows % similarity of 16S rRNA sequences from representative OTUs of the predominating taxa and isolates from each sample. This result corroborated the previous reports of Chelius and Triplett (2001) where there was a 48% overlap of culture-independent and cultivable bacterial communities in the maize roots.

**Table 4:** % similarity of 16S rRNA of representative OTUs (repseq) and culturable isolates.

**Samples**	**Taxa**	**OTU representative sequence**	**% relative abundance**		**16s rRNA isolate strain**	**% similarity (16S rRNA)**
			**16s rRNA amplicons (OTUs)**	**16s rRNA isolate**		
ODC	*Pantoea*		68.8	60		
		Repseq197			O_J13-9	99.2
	*Pseudomonas*		5.3	11		
		Repseq493			O_J13-114	100
	*Xanthomonas*		16			
	*Stenotrophomonas*		1			
	*Rhizobium*		5			
OY	*Pantoea*		7.2			
	*Pseudomonas*		4.1			
	*Xanthomonas*		29	10		
		Repseq592			O_J13-22	100
	*Stenotrophomonas*		7	53		
		Repseq517			O_J13-28	96.2
					O_J13-20	95.1
	*Rhizobium*		7			
		Repseq515			O_J13-35	98.9
	*Curtobacterium*		11.3	3		
		Repseq83			O_J13-29	96.6
					O_J13-21	99.6
	*Paenibacillus*		3.5	8		
		Repseq110			O_J13-18	100
OG	*Pantoea*		96.2	92		
		Repseq197			O_J13-17	99.6
					O_J13-59	99.6
					O_J13-3	99.6
					O_J13-1	99.6
					O_J13-37	99.6
					O_J13-5	99.6
	*Pseudomonas*		1.1	3		
		Repseq493			O_J13-10	97.7
					O_J13-60_	97.7
	Rhizobium*	Repseq515	-	1	O_J13-15	98.9
					O_J13-61	98.9
KDC	*Pantoea*		45.0	32		
	*Pseudomonas*		53.5	9		
		Repseq493			K_J13-129	95.9
	*Xanthomonas*		1	-		
	Serratia*	repseq616	-	48	K_J13-53	96.2
					K_J13-131	95.5
KY	*Pantoea*		63.2	38		
		Repseq197			K_J13-79	99.2
					K_J13-83	99.6
	*Pseudomonas*		7.4	-		
	*Xanthomonas*		1	-		
	*Stenotrophomonas*		1	19		
		Repseq517			K_J13-50	95.1
					K_J13-105	95.1
	Serratia*	repseq616	9.0	-		
KG	*Pantoea*		35.3	-		
	*Pseudomonas*		10.2	55		
		Repseq493			K_J13-89	97.7
					K_J13-109	98.3
	*Xanthomonas*		5	-		
	*Stenotrophomonas*		7	40		
		Repseq517			K_J13-107	95.1
					K_J13-108	95.1
					K_J13-8	94.7
					K_J13-88	94.7
					K_J13-48	95.1
					K_J13-111	95.1
	*Rhizobium*		31	-		
	Serratia*	repseq616	5.4	-		

*% relative abundances of OTUs and isolates per taxa in each sample were also shown*.

Serratia* isolates absent in KG and KY, but present in KDC, still showing >95 % similarity.

Rhizobium* isolates absent in samples with OTU abundance, still showing >95 % similarity when compared with isolates from other samples.

Phosphate is an essential plant nutrient with low bioavailability in soil which is unavailable to the plants. It is well known that improved nutrient uptake of plants is mediated by plant-associated microorganisms. In this study case, the majority of the P solubilizing bacteria were *Pantoea* species.

Indole-3-acetic acid (IAA)—a plant growth hormone, is synthesized by a large number of plant associated bacteria (Long et al., 2008; Merzaeva and Shirokikh, 2010; Khan et al., 2012). In this study, *Pantoea* spp., *Pseudomonas* spp. and *Stenotrophomonas* showed evidence of IAA production. Other bacteria are well known for their production of ACC deaminase. This enzyme is highly influential in the plant environment because it hinders the production of ethylene. Under stressful conditions, production of ethylene is induced; this induction then inhibits plant growth. BEs are known to hinder ethylene biosynthesis through the expression of the enzyme ACC (1-aminocyclopropane-1-carboxylate) deaminase encoded by the *acds* gene that converts the ethylene precursor ACC to α-ketobutyrate and ammonia (Glick, 2005, 2015; Sun et al., 2009; Glick and Stearns, 2011). In this study, a few isolates were found to be putative ACC deaminase producers. These were from species of *Rhizobium, Xanthomonas, Pantoea, Pseudomonas*, and *Stenotrophomonas* from HC contaminated site; and species of *Erwinia, Pseudomonas* and *Pantoea* from non-HC contaminated site.

We expected to find functional differences between the bacterial endophytes isolated from HC and non-HC contaminated sites. However, there was no marked difference on functional capabilities of stem bacterial endophytes isolated from either contaminated or non-contaminated site. Particularly unexpected was virtual absence of known alkane hydroxylase and catechol 2,3-dioxygenase genes in the strain collection. Genomic analyses of *Microbacterium foliorum* 122 and *Plantibacter flavus* 251, bacterial endophytes that are common among the plants in this study, revealed that there were no known genes for classical toluene and naphthalene metabolism despite the fact that both these strains demonstrated utilization of toluene and naphthalene substrates (Lumactud et al., 2017a,b). Further research work is needed to delve deeper into the metabolic characteristics of these endophytes as new hydrocarbon degrading enzymes may possibly be involved.

In summary we found very few differences in adaptive traits in the endophytes of Oil Springs and the control site Komoka. The stem as a habitat may have protected the bacterial endophytes from the stress that is occurring outside the plants, which is likely one of the reasons why there was no marked difference of functional capabilities between contaminated and non-contaminated sites. The selective pressure for the endophytes is more determined by the plant interior than by the exterior contaminants.

## Author contribution

RL conceptualized the project, performed field and laboratory work, analyzed data, wrote the manuscript with supervision and editing by RF.

### Conflict of interest statement

The authors declare that the research was conducted in the absence of any commercial or financial relationships that could be construed as a potential conflict of interest.
